# Public Address on the Intellectual, Moral and Physical Training, Etc.

**Published:** 1854-08

**Authors:** 


*Public Address on the Intellectual, Moral, and Physical Training necessary to constitute the True Physician.* Delivered before the Æsculapian Medical Society, at its Semi-Annual Session, held in Marshall, Illinois, May, 1854. By F. R. PAYNE, M.D. Published by order of the Society.

OUR readers are indebted to the Æsculapian Medical Society for many very interesting articles which have appeared from time to time in this journal. The address of Dr. Payne is only another evidence of the industry and ability of our professional brethren in that section of our state. We have neither space for extracts, nor time for analysis. We thank the Doctor for the happy manner in which he has treated his subject, and solicit for our readers and ourselves the privilege of frequently hearing from him through the pages of our journal. J.

